# Global analysis of more than 50,000 SARS-CoV-2 genomes reveals epistasis between eight viral genes

**DOI:** 10.1073/pnas.2012331117

**Published:** 2020-11-17

**Authors:** Hong-Li Zeng, Vito Dichio, Edwin Rodríguez Horta, Kaisa Thorell, Erik Aurell

**Affiliations:** ^a^New Energy Technology Engineering Laboratory of Jiangsu Province, School of Science, Nanjing University of Posts and Telecommunications, Nanjing, 210023, China;; ^b^Nordic Institute for Theoretical Physics, Royal Institute of Technology and Stockholm University, 10691 Stockholm, Sweden;; ^c^Department of Physics, University of Trieste, 34151 Trieste, Italy;; ^d^Department of Computational Science and Technology, AlbaNova University Center, 10691 Stockholm, Sweden;; ^e^Group of Complex Systems and Statistical Physics, Department of Theoretical Physics, Physics Faculty, University of Havana, 10400 Havana, Cuba;; ^f^Department of Infectious Diseases, Institute of Biomedicine, Sahlgrenska Academy, University of Gothenburg, 40530 Gothenburg, Sweden;; ^g^Center for Translational Microbiome Research, Department of Microbiology, Cell and Tumor Biology, Karolinska Institutet, 17177 Stockholm, Sweden

**Keywords:** SARS-CoV-2, epistasis, recombination, direct coupling analysis

## Abstract

Genome-wide epistasis analysis is a powerful tool to infer gene interactions, which can guide drug and vaccine development and lead to deeper understanding of microbial pathogenesis. We have considered all complete severe acute respiratory syndrome coronavirus 2 (SARS-CoV-2) genomes deposited in the Global Initiative on Sharing All Influenza Data (GISAID) repository until four different cutoff dates, and used direct coupling analysis together with an assumption of quasi-linkage equilibrium to infer epistatic contributions to fitness from polymorphic loci. We find eight interactions, of which three are between pairs where one locus lies in gene ORF3a, both loci holding nonsynonymous mutations. We also find interactions between two loci in gene nsp13, both holding nonsynonymous mutations, and four interactions involving one locus holding a synonymous mutation. Altogether, we infer interactions between loci in viral genes ORF3a and nsp2, nsp12, and nsp6, between ORF8 and nsp4, and between loci in genes nsp2, nsp13, and nsp14. The paper opens the prospect to use prominent epistatically linked pairs as a starting point to search for combinatorial weaknesses of recombinant viral pathogens.

## Significance

The COVID-19 pandemic is a worldwide public health emergency caused by the β-coronavirus SARS-CoV-2. A very large and continuously increasing number of high-quality whole-genome sequences are available. We have investigated whether these sequences show effects of epistatic contributions to fitness. In a population evolving under a high rate of recombination, such effects of natural selection can be detected by direct coupling analysis, a global model learning technique. The paper opens up the prospect to leverage very large collections of genome sequences to find combinatorial weaknesses of highly recombinant pathogens.

The pandemic of the disease COVID-19 has so far led to the confirmed deaths of more than 852,000 people (1) and has hurt millions. As the health crisis has been met by nonpharmacological interventions (2, 3) there has been significant economic disruption in many countries. The search for vaccine or treatment against the new coronavirus severe acute respiratory syndrome coronavirus 2 (SARS-CoV-2) is therefore a worldwide priority. The Global Initiative on Sharing All Influenza Data (GISAID) repository (4) contains a rapidly increasing collection of SARS-CoV-2 whole-genome sequences, and has already been leveraged to identify mutational hotspots and potential drug targets (5). Coronaviruses, in general, exhibit a large amount of recombination (6–9). The distribution of genotypes in a viral population can therefore be expected to be in the state of quasi-linkage equilibrium (QLE) (10–12), and directly related to epistatic contributions to fitness (13, 14). We have determined a list of the largest such contributions from 51,676 SARS-CoV-2 genomes by a direct coupling analysis (DCA) (15, 16). This family of techniques has earlier been used to infer the fitness landscape of HIV-1 Gag (17, 18) to connect bacterial genotypes and phenotypes through coevolutionary landscapes (19) and to enhance models of amino acid sequence evolution (20). We apply a recent enhancement of this technique to eliminate predictions that can be attributed to phylogenetics (shared inheritance) (21). We find that eight predictions stand out between pairs of polymorphic sites located in genes nsp2 and ORF3a, in genes nsp4 and ORF8, and between genes nsp2, nsp6, nsp12, nsp13, nsp14 and ORF3a. Most of these sites have been documented in the literature when it comes to single-locus variations (22–27). The nsp4–ORF8 pair was additionally found to be strongly correlated, in an early study (28). It does not show prominent correlations today, but is ranked second in our global analysis. The epistasis analysis of this paper brings a different perspective than correlations, and highlights pair-wise associations that have remained stable as orders of more SARS-CoV-2 genomes have been sequenced.

## Results

The predicted effective interactions between loci were obtained from pseudo-likelihood maximization (PLM) scores, a standard computational method to perform DCA. Manual inspection shows that about half of the top 50 links and most of the top 200 involve noncoding sites in the 5′ or 3′ region on the “Wuhan-Hu-1” (29) reference sequence, many of them have very short range, and most of them with a large fraction of the gap or N (unknown nucleotide) symbols (data available as Dataset S3 and in ref. 30 for other dataset). We present the links with both terminal loci located in coding regions and the mutations excluding gaps or Ns.

**Fig. 1.**
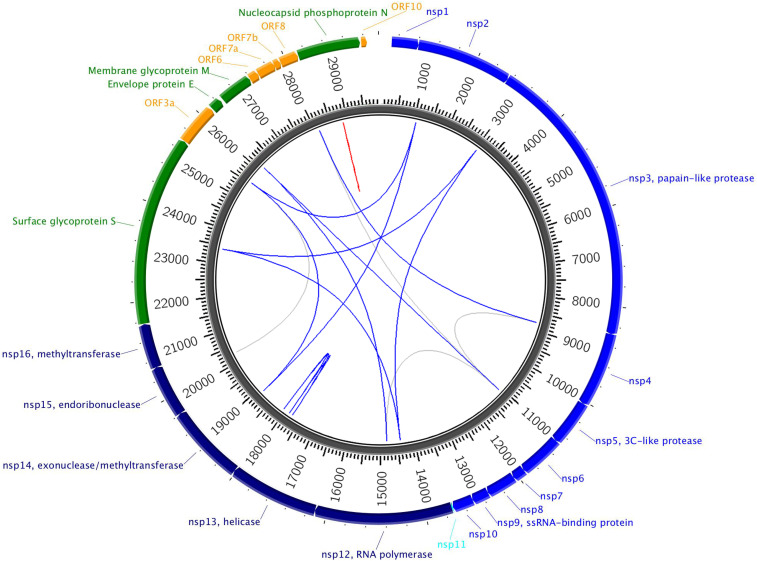
Top 200 significant pairwise epistases from the 8 August 2020 dataset between loci in coding regions. Colored lines indicate top 50, and gray lines indicate top 51 to 200. Red lines show short-distance links (distance less than or equal to 3 bp); blue lines show links of longer distance. The colorful links are the same pairs as listed in Table 1. Analogous circos plots for the 2 May 2020, 1 April 2020, and 8 April 2020 datasets are available in the GitHub repository (30).

In Table 1, we list the significant links for the 8 August 2020 dataset. The first column is the index of each pair-wise interaction in the top 200. The second column indicates the locus with lower genomic position in the pair and the name of the SARS-CoV-2 protein it belongs to. The third column lists the major/minor allele (most prevalent, second most prevalent nucleotide) and the mutation type at that locus. The following two columns provide similar information on the locus with higher genomic position in the pair. The last column contains the PLM scores indicating the strength of effects between pairs of loci. The pair-wise epistases listed in Table 1 for 8 August 2020 dataset are visualized by circos software in Fig. 1, where the red is for the close effects (the distance between two loci is less than or equal to three loci), while blue is for distant effects. Analogous results for the 2 May 2020 dataset is listed in *SI Appendix*, Table S6, and for the 1 April 2020 and 8 April 2020 datasets in ref. 30.

**Table 1.** Significant links with rank within the top 200 between pair-wise loci for the 8 August 2020 dataset

	Locus 1		Locus 2		
Rank*	Protein^†^	Mutation type^‡^	Protein	Mutation type	PLM score
1	1059-nsp2	C| T-non.	25563-ORF3a	G| T-non.	1.7191
2	28882-N	G| A-syn.	28883-N	G| C-non.	1.4996
3	28881-N	G| A-non.	28882-N	G| A-syn.	1.4816
4	28881-N	G| A-non.	28883-N	G| C-non.	1.4783
5	8782-nsp4	C| T-syn.	28144-ORF8	T| C-non.	1.4471
9	14805-nsp12	C| T-syn.	26144-ORF3a	G| T-non.	1.1392
12	3037-nsp3	T| C-syn.	14408-nsp12	T| C-non.	1.0291
13	18877-nsp14	C| T-syn.	25563-ORF3a	G| T-non.	1.0131
14	3037-nsp3	T| C-syn.	23403-S	G| A-non.	1.0114
17	14408-nsp12	T| C-non.	23403-S	G| A-non.	0.9917
21	1059-nsp2	C| T-non.	18877-nsp14	C| T-syn.	0.9197
26	17858-nsp13	A| G-non.	18060-nsp14	C| T-syn.	0.8624
27	17747-nsp13	C| T-non.	17858-nsp13	A| G-non.	0.8553
36	17747-nsp13	C| T-non.	18060-nsp14	C| T-syn.	0.7780
47	11083-nsp6	G| T-non.	26144-ORF3a	G| T-non.	0.7340
63	20268-nsp15	A| G-syn.	25563-ORF3a	G| T-non.	0.6474
134	11083-nsp6	G| T-non.	14805-nsp12	C| T-syn.	0.5040
147	11083-nsp6	G| T-non.	28144-ORF8	T| C-non.	0.4928
168	8782-nsp4	C| T-syn.	11083-nsp6	G| T-non.	0.4770

* Indices of significant links in the top 200 with both terminals located inside a coding region, inferred by PLM. The analogous table for the 2 May 2020 dataset is shown in *SI Appendix*, Table S6.

† Information on locus 1 includes index in the reference sequence, and the protein it belongs to. The convention used is that locus 1 (“starting locus”) is the locus of lowest genomic position in the pair.

‡ Information on mutations of locus 1 includes the first and second prevalent nucleotide at this locus, and mutation type: synonymous(syn.)/nonsynonymous(non.).

**Fig. 2.**
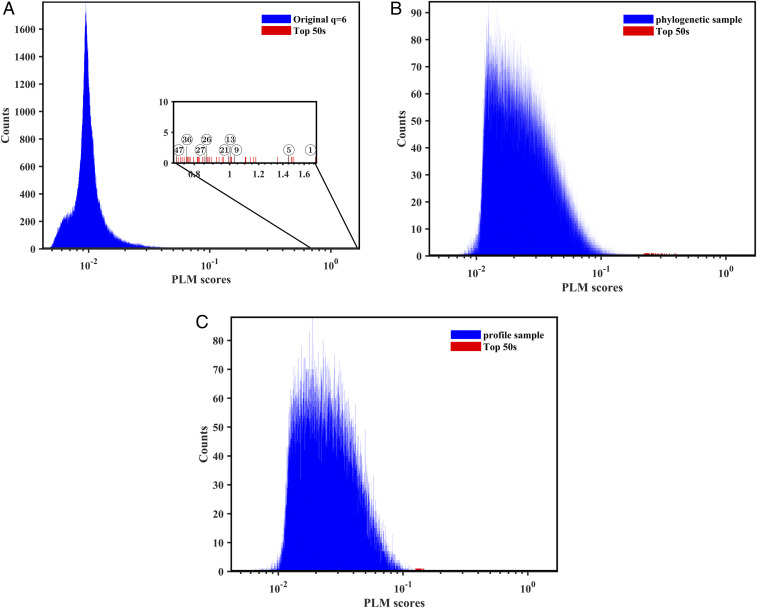
Histograms of PLM scores for (*A*) original 8 August 2020 dataset, (*B*) a phylogenetic randomized sample, and (*C*) a profile randomized sample. The blue bars are for all scores, while the red ones are for the top 50 largest scores. Red arrows in *A* indicate links listed in Table 3. The largest PLM score is pointed to by red arrows for random samples in *B* and *C*. None of them is located inside a coding region, and none of them appear in Tables 1 and 3.

To check whether the interactions can be explained by phylogeny (inherited variations), we used two randomization strategies, “profile” and “phylogeny” of the multiple sequence alignments (MSAs). Profile preserves the distribution over alleles at every locus but does so independently at each locus. Profile hence destroys all systematic covariations between loci. Phylogeny additionally preserves the genetic distance between each pair of sequences. Viral genealogies inferred from this information are therefore unchanged under this randomization. PLM scores run on these two types of randomized data (scrambled MSAs) are a background from which the significance of the interactions from the original data can be assessed. Each randomization strategy is repeated 50 times with different realizations of the scrambling; see *SI Appendix*, Figs. S1–S3 and ref. 30. As shown in Fig. 2, the distribution of PLM scores using phylogeny and profile are qualitatively different from PLM scores of the original MSA, with progressively fewer interactions at high score values. With profile randomization, no interactions predicted by PLM appear with scores standing out from the background. Phylogeny randomization, on the other hand, preserves some interactions found by PLM in a fraction of the realizations of the random background. Table 2 lists interactions predicted by PLM that appear in some phylogeny randomizations with scores large compared to the background. In the following analysis, we have not retained them; see *SI Appendix*, Figs. S1–S3 for circos visualizations. Table 3 lists the eight interactions found by PLM which either do not appear in any phylogeny randomization with scores that stand out from the background, or, in the case of (1059–25563), shows up three times in the top 200 out of 50 samples. We retain these eight predicted epistatic interactions in the sampled populations of SARS-CoV-2 genomes. The top ones listed in Table 3 are marked by red bars in Fig. 2*A*.

**Table 2.** Top 200 that appeared (with an appearance ratio ≥10%) in samples with phylogeny randomization strategy based on the 8 August 2020 dataset

	Locus 1		Locus 2	
Appearance ratio*, %	Protein	Mutation type	Protein	Mutation type
14	28881-N	G| A-non.	28882-N	G| A-syn.
16	28881-N	G| A-non.	28883-N	G| C-non.
20	28882-N	G| A-syn.	28883-N	G| C-non.
22	3037-nsp3	T| C-syn.	14408-nsp12	T| C-non.
20	3037-nsp3	T| C-syn.	23403-S^†^	G| A-non.
34	14408-nsp12	T| C-non.	23403-S^†^	G| A-non.

Fifty phylogeny samples are considered in total.

* The indices of samples with phylogeny randomization which preserve the links listed in Table 1 are shown here. The circos plots for the significant epistatic links of all 50 randomized samples are available in SI Appendix.

† In amino acid notation, this mutation is D614G in Spike.

**Table 3.** Potentially significant epistatic links in Table 1, and corresponding amino acid mutations

	Locus 1		Locus 2	
Rank*	Protein	Amino acid mutation	Protein	Amino acid mutation
1^†^	1059-nsp2	T85I(T^‡^)	25563-ORF3a	Q57H(Q)
5	8782-nsp4	S76S(S)	28144-ORF8	L84S(L)
9	14805-nsp12	T455I(Y)	26144-ORF3a	G251V(G)
21	1059-nsp2	T85I(T)	18877-nsp14	L280L(L)
26	17858-nsp13	T541C(Y)	18060-nsp14	L7L(L)
27	17747-nsp13	P504L(P)	17858-nsp13	T541C(Y)
36	17747-nsp13	P504L(P)	18060-nsp14	L7L(L)
47	11083-nsp6	L37F(L)	26144-ORF3a	G251V(G)

* Main prediction: eight epistatic links. The links preserved by phylogeny randomization in Table 2 are not listed here.

† This link appears in 3 out of 50 (6%) phylogeny randomizations; once (experiment 23) with rank 34, and twice (experiments 29 and 47) with ranks in 51 to 200; see *SI Appendix*, Figs. S2 and S3.

‡ Amino acid in the reference sequence Wuhan-Hu-1 at the positionspecified by the number between major and minor alleles.

**Fig. 3.**
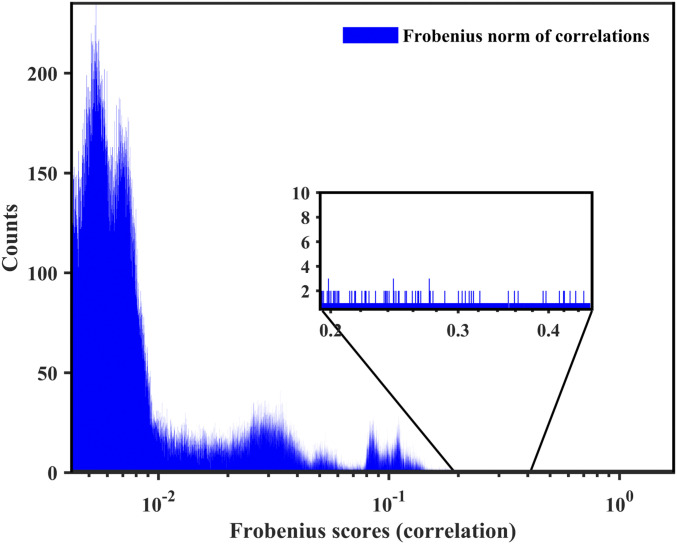
Frobenius norm of pair-wise correlations between loci for the original 8 August 2020 dataset. The score pointed by the red arrow corresponds to the link of 1059 and 25563.

**Fig. 4.**
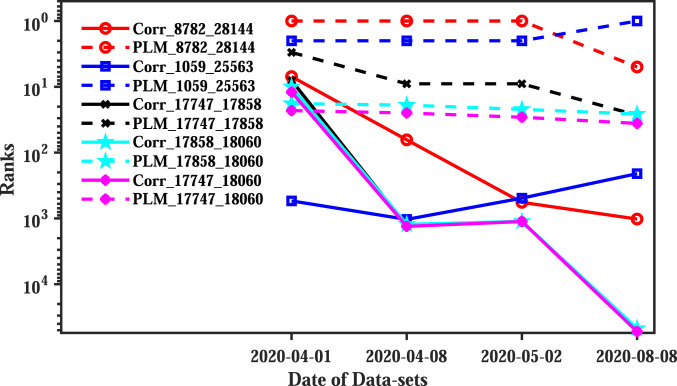
Ranks for significant epistatic effects with data collection date (1 April 2020, 8 April 2020, 2 May 2020, and 8 August 2020) by PLM (dashed lines) and correlation analysis (solid lines). The ranks of the PLM scores are almost constant, while the ranks of the correlations vary significantly and mostly drop as more data accumulate (later cutoff dates).

Epistatic interactions obtained from DCA reflect pair-wise statistical associations, but not correlations. As reviewed in ref. 31, and described in *SI Appendix*, *Methods of DCA*, DCA is based on a global probabilistic model, and therefore ranks interdependency differently than correlations. Fig. 3 compared to Fig. 2 shows that the distribution of correlation scores is qualitatively different from the distributions of DCA scores in the GISAID dataset. Fig. 4 further shows that the ranks of the epistatic interactions predicted in Table 3 have remained stable, while the corresponding correlations have merged into the background.

The first-ranked interaction between 1059 and 25563 is between a (C/T), resulting in the T85I nonsynonymous mutation in gene nsp2, and a (G/T), resulting in the Q57H nonsynonymous mutation in gene ORF3a. The nsp2, expressed as part of the ORF1a polyprotein, binds to host proteins prohibitin 1 and prohibitin 2 (PHB1 and PHB2) in SARS-CoV (32). The variations in site 1,059 have been predicted to modify nsp2 RNA secondary structure (33) and have previously been reported to cooccur together with the Q57H variant in ORF3a in a dataset of SARS-CoV-2 genomes from the United States (34). ORF3a, also known as ExoN1 hypothetical protein sars3a, forms a cation channel of which the structure in SARS-CoV-2 is known by cryoelectron microscopy (cryo-EM) (35). In SARS-CoV, ORF3a been shown to up-regulate expression of fibrinogen subunits FGA, FGB, and FGG in host lung epithelial cells (36), to form an ion channel which modulates virus release (37), and to activate the NLRP3 inflammasome (38), and has been found to induce apoptosis (39). The Q57H variant was reported early in the COVID-19 pandemic (40) and occurs in the first transmembrane alpha helix, TM1 (35), where it changes the amino acid glutamine (Q) with a noncharged polar side chain to histidine (H), which has a positively charged polar side chain. This amino acid is at the interface of interaction between the two dimeric subunits of ORF3a that forms the constrictions of the ion channel, but the Q57H alteration does not seem to change the ion channel properties compared to wild-type 3a (35). Nevertheless, its incidence is increasing in SARS-CoV-2 genomes in the United States (34), and the effect of Q57H may therefore affect the virulence in other beneficial ways than changing the conductance properties of the ion pore.

The association between 8782 and 28144 (rank 5), reported early in SARS-CoV-2 studies (28), is between a (C/T) synonymous mutation in the gene nsp4, and a (T/C) nonsynonymous mutation resulting in the L84S alteration in the gene ORF8. The first of these genes participates in the assembly of virally induced cytoplasmic double-membrane vesicles necessary for viral replication. The site 8782 is located in a region annotated as CpG rich and is the site of a CpG for the major allele (C); it has the minor (T) allele in other related viruses (28). Orf8 has been implicated in regulating the immune response (41, 42). The L84S variant is, together with the C8782T nsp4 mutation, characterizing the GISAID clade S (43).

The interaction between 14805 and 26144 (rank 9) leads to nonsynonymous alterations in nsp12 (T455I; note that the reference is Y) and ORF3a (G251V), respectively. The G251V has been reported by many studies and is defining the GISAID V clade (43) together with the L37F nsp6 variant (position 11083, rank 47). The widely reported G251V variant is, unfortunately, outside of the proposed cryo-EM structure (35), and it is unknown how this glycine to valine substitution affects protein function. The nsp12 is the RNA-dependent RNA polymerase, and the T455I substitution is found where the reference Wuhan-Hu-1 has a tyrosine residue in one of the alpha helices of the polymerase “finger” domain (44). Threonine can, similarly to tyrosine, be phosphorylated but also glycosylated, it is polar and uncharged, and it can form hydrogen bonds that may stabilize the alpha helix. Isoleucine, on the other hand, is nonpolar and uncharged, and both the residues are smaller than the aromatic tyrosine.

The second interaction partner of G251V is the nsp6 L37F variant. The nsp6 has been shown to induce autophagosomes in the host cells in favor for viral replication and propagation SARS-CoV (32). There is currently no experimentally validated model of nsp6 structure, but an early model suggests that the L37P variant is situated in an unordered loop between two alpha helices (45).

The interaction between 17747 and 17858 (rank 27) is between two nonsynonymous mutations (C/T, resulting in P504L) and (A/G, resulting in T541C) within the gene nsp13 that codes for a helicase enzyme that unwinds duplex RNA (32). It is the only epistatic interaction in Table 3 within one protein. These same two loci reappear in the list with ranks 26 and 36 as interacting with a C/T synonymous mutation (L7L) in gene nsp14 at position 18060. The P504L and T541C are both located in the Rec2A part of the protein that is not in direct interaction with the other members of the RNA-dependent RNA polymerase holoenzyme, in which two molecules of nsp13 form a stable complex with nsp12 replicase, nsp7, and nsp8. The nsp14 protein is a bifunctional protein that has an N7-methyltransferase domain and a domain exonuclease activity, responsible for replication proofreading (46). The nsp14/nsp10 proofreading machinery is thought to interact with the replication–transcription complex, but the exact details of this interaction are not known.

The final interaction (rank 21) is a link between a locus carrying a nonsynonymous mutation (C/T, T541C) in nsp2 position 1059 and a locus carrying a synonymous mutation (C/T, L280L) in nsp14, position 18877. As the knowledge on nsp2 protein structure is poor, there is no evidence for the effect of this mutation. Also, how the synonymous C/T alterations in nsp14, as well as in the synonymous mutations of the other interactions, affect the virus is unknown, but can be proposed to change RNA secondary structure, RNA modification, or codon usage.

## Discussion

In this work, we have considered all whole-genome sequences of SARS-CoV-2 deposited in GISAID up to different cutoff dates. As this coronavirus has extensive recombination, we have assumed that the distribution of genotypes is well described by Kimura’s QLE, and used DCA to infer epistatic contributions to fitness from the sequences. After filtering out all but the strongest effects and variations in noncoding regions with many gaps in the MSA, the remaining predictions are few in number, i.e., 19 predictions in Table 1.

Covariations between allele distributions at different loci can be due to epistasis and also to inherited effects (phylogeny). We have tested for the second type by randomizing MSA of sequences such that pair-wise distances between sequences are left invariant. We find that the top link 1059–25563 appears three times in 50 phylogeny randomizing samples, although with much lower rank. The other predicted epistatic contributions disappear under phylogenetic randomization, except for pairs in the triple (3037, 14408, 23403) which appear in from 20 to 35% of 50 randomizations. After eliminating these links as well as links between adjacent loci (28881, 28882, 28883, which appear in from 14 to 16% in 50 samples), we are left with eight predictions listed in Table 3. We consider it likely that these retained interactions are due to epistasis, and not to inherited covariation. An analogous investigation on a smaller dataset obtained with an earlier cutoff date (2 May 2020) and reported in *SI Appendix*, Tables S6 and S7 and Fig. S6 yielded six retained predictions, involving, however, the same eight viral genes. The question on epistasis vs. effects of inheritance (phylogeny) clearly merits further investigation and testing as more data become available.

Biological fitness is a many-sided concept and can also include aspects of game and cooperation (47–49). A fitness landscape describes the propensity of an individual to propagate its genotype in the absence of strategic interactions with other genotypes, and has traditionally been used to model the evolution of pathogens colonizing a host; for earlier use relating to HIV and using DCA techniques, see ref. 50. The additive and epistatic contributions to fitness of the virus which we find describe the virus in its human host and therefore likely reflect host–pathogen interactions to a large extent. A conceptual simplification made is that all hosts have been assumed equivalent. In future methodological studies, it would be of interest to consider possible effects of evolution in a collection of landscapes, representing different hosts, and to correlate such dynamics to host genotypes. As this requires other data than are available on GISAID, and which are less abundant at this time, we leave this for future work. On the other hand, it is unlikely that the inferred couplings involve the host as a temporal variable, due to the much faster time scale of the evolution of the virus.

Epistatic interactions are pair-wise statistical associations, but are not simply correlations. The interaction between sites 8782 and 28144, which is the second largest in Table 3, was identified as a very strong correlation in a very early study (28). As shown in Table 4, this correlation has generally decreased over time (using data with successively later cutoff dates). In the alternative global model learning method of DCA which we use in the present work, the score of statistical interdependency of this pair has remained large, and the pair is consistently ranked first or second over four different cutoff dates; see Fig. 4. While our data hence support the observation of statistical interdependency in this pair first made in ref. 28, they do not support the interpretation made in the same work that the effect is due to phylogeny. The later criticism in ref. 51 therefore does not apply to our work, since an epistatic interaction, recovered through DCA and a QLE assumption in a population thoroughly mixed by recombination, is different in nature from a phylogenetic effect.

**Table 4.** Top 10 links found by correlation analysis in the coding region for the dataset until 8 August 2020

Rank*	Locus 1 protein	Locus 2 protein	Frobenius score
455	3037-nsp3	23403-S	0.3844
458	3037-nsp3	14408-nsp12	0.3842
460	14408-nsp12	23403-S	0.3837
581	28882-N	28883-N	0.3609
584	28881-N	28883-N	0.3603
585	28881-N	28882-N	0.3603
1071	1059-nsp2	25563-ORF3a	0.2821
2394	8782-nsp4	28144-ORF8	0.1803
3969	23403-S	28144-ORF8	0.1487
3980	3037-nsp3	28144-ORF8	0.1486

* Rank for top 10 links as ranked by correlation analysis. Correlations between loci of which at least one is outside coding regions are omitted.

DCA techniques have been applied to find candidate targets for vaccine development. In a series of studies, it was found that combinations of mutations implied by sequence variability in the HIV-1 Gag protein correlate well with in vitro fitness measurements, and with clinical observations on escape strains (HIV strains that tend to dominate in one patient over time) and the immune system of elite controllers (HIV-positive individuals progressing slowly toward AIDS) (18, 50, 52). While this may be a promising future avenue in COVID-19 research, in the present study, we have not found any epistatic interactions involving Spike, only pairs that also show up under phylogeny randomization or that are quite weak; see *SI Appendix*, Table S4. The Spike protein has been the main target of coronavirus vaccine development to date (53), including against SARS-CoV-2 (54–56).

An epistatic interaction means that loss of fitness by a mutation at one locus is enhanced (positive epistasis) or compensated (sign epistasis) by a mutation at another locus. Suppose there are drugs that act on targets around both loci, modulating the fitness of the respective variants. Epistasis then points to the possibility that using both drugs simultaneously may have a more than additive effect. To search whether our analysis offers such a guide to combinatorial drug treatment, we scanned the recent comprehensive compilation of drugs known or predicted to target SARS-CoV-2 (57). Five out of the eight predictions in Table 3 involve either one synonymous mutation or are between two mutations in the same gene. For all of the three remaining pairs of nonsynonymous mutations, (1059, 25563), (11083, 26144), and (14805, 26144), the second locus lies in ORF3a, for which no potential drugs are listed in ref. 57. The first locus in the same three pairs lies, respectively, in genes nsp2, nsp6, and nsp12. One or more already approved and practical drugs targeting nsp2 and nsp6 are listed in ref. 57. Ponatinib, listed for nsp12, is not appropriate against a pandemic disease like COVID-19 on account of its large cost. Potential drugs for the proteins listed in Table 3 are summarized in *SI Apppendix*, Table S5, following ref. 57.

Nevertheless, the number of combinations of potential drug targets, in COVID-19 and many other diseases, is very large. DCA applied to many sampled sequences predicts which genes/loci have mutual dependencies in fitness, and can be used to rank combinations for further, more detailed investigation. We note that one can also start a search for drug treatment from conserved positions, assuming these to be unconditionally necessary for the virus. If so, all potential pairs would, however, be ranked equal based on sample information, and there would be no analogous shortcut to the combinatorial explosion of possibilities. Even if the scan discussed above did not lead to any direct suggestions based on the lists of potential drugs in ref. 57, we hope the general approach could have value given the continuing increase and availability of genome sequences of both viral and bacterial pathogens. We finally note that three out of eight of our list of predictions involve loci in viral gene ORF3a, the action of which is related to severe manifestations of COVID-19 disease (37–39).

## Materials and Methods

### Data.

We analyzed the consensus sequences deposited in the GISAID database (4) with high quality and full lengths (number of bps ≈30,000). Four datasets are used for our investigation according to the collection date in GISAID database. The dates are 1 April 2020, 8 April 2020, 2 May 2020, and 8 August 2020. The list of GISAID sequences used is given in Dataset S1, and is also available on the Github repository (30). The numbers of selected genomes are 2,268, 3,490, 10,587, and 51,676 for the respective collection dates.

### MSA.

MSAs were constructed with the online alignment server Multiple sequence alignment using Fast Fourier Transform (MAFFT) (58, 59) for the two smaller datasets with cutoff dates 1 April 2020 and 8 April 2020. To align the two larger datasets with more than 10,000 sequences, a prealigned reference MSA is recommended to accelerate the alignment and reduce the burden on computational resources. Here, we took the collection with cutoff date 8 April 2020 as the prealigned reference MSA for the two largest datasets with cutoff dates 2 May 2020 and 8 August 2020. The MSAs used are given as Dataset S2, and are also available on the Github repository (30).

The MSA is a big matrix $S=\left\{\sigma_{i}^{n}|i=1,\ldots ,L,n=1,\ldots ,N\right\}$, composed of N genomic sequences which are aligned over L positions (16, 21). Each entry $\sigma_{i}^{n}$ of matrix S is either one of the four nucleotides (A, C, G, T), or “not known nucleotide” (N), or the alignment gap “-” introduced to treat nucleotide deletions or insertions, or some minorities.

### MSA Filtering.

Before filtering, we transform the MSA in two different ways as follows: 1) The gaps “-” are transformed to “N” while the minors “KFY…” are mapped to “N.” Thus five states remain, where “NACG”’ are represented by “12345”; 2) the minors “KFY…” are mapped to “N.” Then there are six states, with “-NACGT” represented by “012345.”

The following criteria are used for prefiltering of the MSA from the 8 August 2020 dataset. If, for one locus, the same nucleotide is found in more than 96.5% of this column, or if the sum of the percentages of A, C, G, and T at this position is less than 20%, then this locus will be deleted. For each sequence, if the percentage of a certain nucleotide is more than 80%, or if the sum of the percentages of A, C, G, and T in this sequence is less than 20%, then this sequence will be deleted. With this filtering criteria, many loci but no sequences are deleted. There are left 51,676 sequences and 689 loci.

### B-effective Number.

To mitigate the effects of dependent samplings, it is standard practice to attach to each collected genome sequence $\sigma^{\left(b\right)}$ a weight $w_{b}$ (15, 16, 60), which normalizes its impact on the inference procedure. An efficient way to measure the similarity between two sequences $\sigma^{\left(a\right)}$ and $\sigma^{\left(b\right)}$ is to compute the fraction of identical nucleotides and compare it with a preassigned threshold value x in the range 0≤x≤1. The weight of a sequence $\sigma^{\left(b\right)}$ can be set as $w_{b}=1/m_{b}$, with $m_{b}$ as the number of sequences in the MSA that are similar to $\sigma^{\left(b\right)}$,

$$m_{b}=|\left\{a\in \left\{1,\ldots ,B\right\}\right\}:overlap\left(\sigma^{\left(a\right)},\sigma^{\left(b\right)}\right)\geq x|;$$ [1]

here, overlap is the fraction of loci where the two sequences are identical. The B-effective number of the transformed sequences is defined as

$$B_{e\mathrm{ff}}=\overset{B}{\underset{b=1}{\sum }}w_{b}.$$ [2]

**Fig. 5.**
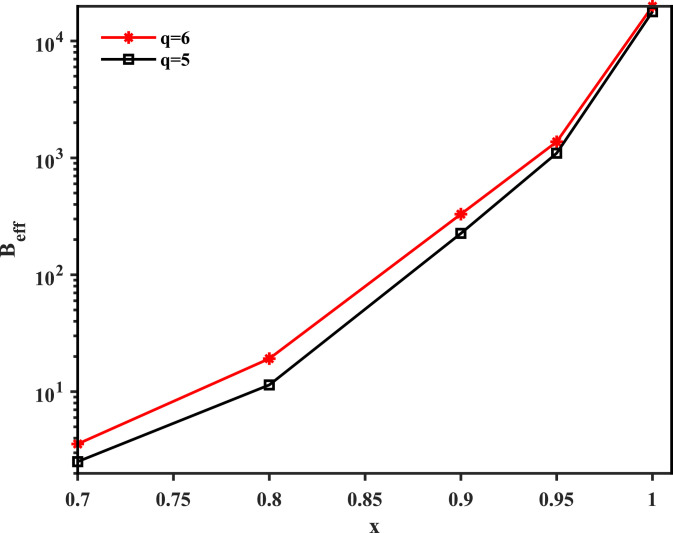
$B_{e\mathrm{ff}}$ number of the 8 August 2020 prefiltered dataset with threshold x. Red denotes q=6 states (“-NACGT”), and black denotes q=5 states (“NACGT”). The number of states is determined by the transform criteria of the prefiltered MSA.

We compare the $B_{e\mathrm{ff}}$ value with different x for the filtered MSA with q=5 and q=6. As shown in Fig. 5, the dataset with six states shows larger $B_{e\mathrm{ff}}$ number for all tested x. We thus perform our analysis on the dataset with q=6 states, where “-NACGT” is represented by “012345.”

The reweighting procedure partially addresses a point raised (51), that sequenced viral genomes are not a random sample of the global population. That is, even if sequencing is biased by the country they occur in and by contact tracing, sufficiently similar genomes will have lower weight, and so each will contribute less to predictions.

### Elements of QLE.

The phenomenon of QLE was discovered by M. Kimura (10) while investigating the steady-state distribution over two biallelic loci evolving under mutation, recombination, and selection, with both additive and epistatic contributions to fitness. In the absence of epistasis, such a system evolves toward linkage equilibrium (LE) where the distribution of alleles at the two loci are independent. The covariance of alleles at the two loci then vanishes. In the presence of pair-wise epistasis and sufficiently high rate of recombination, the steady-state distribution takes form of a Gibbs–Boltzmann form

$$P\left(\sigma_{1},\ldots ,\sigma_{L}\right)=\frac{1}{Z}\exp\left\{-H\left(\sigma_{1},\ldots ,\sigma_{L}\right)\right\},$$ [3]

with an “energy function”

$$H\left(\sigma_{1},\ldots ,\sigma_{L}\right)=\underset{i}{\sum }h_{i}\left(\sigma_{i}\right)+\underset{ij}{\sum }J_{ij}\left(\sigma_{i},\sigma_{j}\right).$$ [4]

In the above, $J_{ij}$ can be related to the epistatic contribution to fitness between loci i and j with alleles $\sigma_{i}$ and $\sigma_{j}$ (11–13). The quantity $h_{i}$ is similarly a function of allele $\sigma_{i}$ which depends on both additive and epistatic contributions to fitness involving locus i. It has been verified in in silico testing that, when the terms in Eq. **4** can be recovered, this is a means to infer epistatic fitness from samples of genotypes in a population (14). In the bacterial realm, this approach was used earlier to infer epistatic contributions to fitness in the human pathogens *Streptococcus pneumonia* (61) and *Neisseria gonorrhoeae* (62), both of which are characterized by a high rate of recombination. The method was also tested on data on the bacterial pathogen *Vibrio parahemolyticus* (63). In that study, the results from DCA were not superior to an analysis based on Fisher exact test; see *SI Appendix*, *Different Quantifications of Correlations* for a discussion. This is consistent with the approach taken here, as *V. parahemolyticus* has a low rate of recombination. Further details on the QLE state of evolving populations are given in *SI Appendix*, *Quasi-Linkage Equilibrium (QLE) and Its Range of Validity*.

### Inference Method for Epistasis between Loci.

The basic assumption of modeling the filtered MSA is that it is composed of independent samples that follow the Gibbs–Boltzmann distribution Eq. **3** with H as in Eq. **4**. Higher-order interactions are also possible to include, but we ignore them here (64). This assumption is a simplification of the biological reality; however, it provides an efficient way to extract information from massive data.

On the other hand, in the context of inference from protein sequences, it has been argued that the one encoded in Eqs. **3** and **4** is the minimal generative model, that is, capable not only of reproducing the empirical frequencies and correlations but also of generating new sequences indistinguishable from natural sequences (16, 65, 66).

Many techniques have been developed to infer the direct couplings in Eq. **3**, as reviewed in ref. 31 and references therein; see also *SI Appendix*, *Methods of DCA*. We employ the PLM method (13, 60, 67–70) to infer the epistatic effects between loci from the aligned MSA. PLM estimates parameters from conditional probabilities of one sequence conditioned on all of the others. For a Potts model with multiple states q>2, this conditional probability is

$$P\left(\sigma_{i}|\sigma_{\backslash i}\right)=\frac{\exp\left(h_{i}\left(\sigma_{i}\right)+\sum_{j\neq i}J_{ij}\left(\sigma_{i},\sigma_{j}\right)\right)}{\sum_{u}\exp\left(h_{i}\left(u\right)+\sum_{j\neq i}J_{ij}\left(u,\sigma_{j}\right)\right)},$$ [5]

with u={0,1,2,3,4,5} as the possible state of $\sigma_{i}$. Eq. **5** depends on a much smaller parameter set compared with that in Eq. **3**. This leads to a much faster inference procedure of parameters compared with the maximum likelihood method. With a given independent sample set, one can maximize the corresponding log-likelihood function

$$\begin{array}{cc} PL_{i}\left(h_{i},\left\{J_{ij}\right\}\right) & =\frac{1}{N}\underset{s}{\sum }h_{i}\left(\sigma_{i}^{\left(s\right)}\right)+\frac{1}{N}\underset{s}{\sum }\underset{j\neq i}{\sum }J_{ij}\left(\sigma_{i}^{\left(s\right)},\sigma_{j}^{\left(s\right)}\right)\\ & -\frac{1}{N}\underset{s}{\sum }\log\underset{u}{\sum }\exp\left(h_{i}\left(u\right)+\underset{j\neq i}{\sum }J_{ij}\left(u,\sigma_{j}^{\left(s\right)}\right)\right) \end{array},$$ [6]

where s labels the sequences (samples), from 1 to N. With the filtered MSA, we then run the asymmetric version of PLM (60) in the implementation PLM available in ref. 71 with regularization parameter λ=0.1. The inferred interactions between loci i and j are scored by the Frobenius norm.

### Relation to Correlation Analysis.

In LE, the distributions of alleles over different loci are independent. Given unlimited data and unlimited computational resources, the terms $J_{ij}$ in Eq. **4** inferred from the data would then be zero. The locus–locus covariances, defined as

$$c_{ij}\left(a,b\right)=\left\langle 1_{\sigma_{i},a}1_{\sigma_{j},b}\right\rangle -\left\langle 1_{\sigma_{i},a}\right\rangle \left\langle 1_{\sigma_{j},b}\right\rangle ,$$ [7]

would also be zero. The Frobenius norm of $c_{ij}\left(a,b\right)$ over indices (a,b) as a score of strength of correlations would be zero as well. The qualitative difference between correlation analysis and global model inference based on Eqs. **3** and **4** is that two loci i and j may be correlated (“indirectly coupled”) even if their interaction $J_{ij}$ is zero, provided they both interact with a third locus k. Data in Table 4 and Fig. 4 show that the leading interactions retrieved by DCA cannot be stably recovered in correlation analysis. A different score of statistical dependency between two categorical random variables is mutual information (MI). Results in *SI Appendix*, Fig. S5 and Table S1 show that the result does not substantially change if using MI instead of Frobenuis norm of correlation matrices. Circos plots of interactions based on correlation scores are available in ref. 30.

### Epistasis Analysis with PLM Scores.

PLM procedure yields a fully connected paradigm between pair-wise loci. To extract important information from massive SARS-CoV-2 genomic sequences, we focus on the significant scores between loci, the top 200 pairs. With a reference sequence “Wuhan-Hu-1,” we identify the positions of the corresponding nucleotides. The visualization of these epistases is performed by “circos” software (72).

### Randomized Background Distributions.

A way to assess the validity of a small number of leading retained predictions among a much larger set of mostly discarded predictions is to compare to randomized backgrounds. The retained predictions are then, in any case, large (by some measure) and would also be retained if selection were made according to some cutoff, or an empirical p value. The problem is thus how to distinguish the case where a small subset of retained values are large, because they are different, from the case when, in a large number of samples, such values would appear at random. This problem can be addressed by comparing the retained values to the largest values from the same procedure applied to randomized data, as was done for predicted RNA–RNA binding energies in a noncoding RNA discovery pipeline (73). In the context of DCA (PLM) applied to genome-scale MSAs, two earlier studies relying on randomized background distributions are described in refs. 13 and 74.

### PLM Scores with Randomization.

To understand the nature of the top 200 PLM scores, we perform two distinct randomization strategies on the MSA, such that its conservation patterns and (or) phylogenetic relations are preserved, while intrinsic coevolutionary couplings (epistatic interactions) are removed (75). Running DCA on artificial sequences ensembles generated by these strategies, and comparing them to the results obtained from original MSA, allows disentangling of spurious couplings given by finite-size effects or by phylogeny. The first strategy, which we refer to as “profile,” randomizes the input MSA by random but independent permutation of all its columns, conserving the single-column statistics for all sites. This destroys all kind of correlations, and DCA couplings inferred from such samples are only nonzero due to the noise caused by finite sample size. In the second strategy, referred to as “phylogeny,” the original MSA is randomized by a simulated annealing schedule where columns and rows are changed simultaneously but so that intersequence distances are kept invariant. Phylogeny inferred from intersequence distance information would therefore be unchanged. Conversely, if the predicted epistatic interactions are due to phylogeny, they should also show up in terms recovered by PLM from MSAs scrambled by “phylogeny.” More details on the randomization strategies can be found in *SI Appendix*, *Phylogenetic Randomization of DCA: Principles*.

## Supplementary Material

Supplementary File

Supplementary File

Supplementary File

Supplementary File

## Data Availability

All study data are included in the article and *SI Appendix*.
